# Association of Cell Adhesion Molecules Contactin-6 and Latrophilin-1 Regulates Neuronal Apoptosis

**DOI:** 10.3389/fnmol.2016.00143

**Published:** 2016-12-15

**Authors:** Amila Zuko, Asami Oguro-Ando, Harm Post, Renske L. R. E. Taggenbrock, Roland E. van Dijk, A. F. Maarten Altelaar, Albert J. R. Heck, Alexander G. Petrenko, Bert van der Zwaag, Yasushi Shimoda, R. Jeroen Pasterkamp, J. Peter H. Burbach

**Affiliations:** ^1^Department of Translational Neuroscience, Brain Center Rudolf Magnus, University Medical Center UtrechtUtrecht, Netherlands; ^2^Biomolecular Mass Spectrometry and Proteomics, Bijvoet Center for Biomolecular Research and Utrecht Institute for Pharmaceutical Sciences, Utrecht UniversityUtrecht, Netherlands; ^3^Netherlands Proteomics CentreUtrecht, Netherlands; ^4^Laboratory of Receptor Cell Biology, Shemyakin-Ovchinnikov Institute of Bioorganic Chemistry Russian Academy of SciencesMoscow, Russia; ^5^Department of Genetics, University Medical Center UtrechtUtrecht, Netherlands; ^6^Department of Bioengineering, Nagaoka University of TechnologyNagaoka, Japan

**Keywords:** autism, ASD, neurodevelopmental disorders, cell adhesion molecules, neuronal outgrowth, Cntn6, Lphn1, apoptosis

## Abstract

In view of important neurobiological functions of the cell adhesion molecule contactin-6 (Cntn6) that have emerged from studies on null-mutant mice and autism spectrum disorders patients, we set out to examine pathways underlying functions of Cntn6 using a proteomics approach. We identified the cell adhesion GPCR latrophilin-1 (Lphn1, a.k.a. CIRL1/CL, ADGRL1) as a binding partner for Cntn6 forming together a heteromeric *cis*-complex. Lphn1 expression in cultured neurons caused reduction in neurite outgrowth and increase in apoptosis, which was rescued by coexpression of Cntn6. In cultured neurons derived from *Cntn6^-/-^* mice, Lphn1 knockdown reduced apoptosis, suggesting that the observed apoptosis was Lphn1-dependent. In line with these data, the number of apoptotic cells was increased in the cortex of *Cntn6^-/-^* mice compared to wild-type littermate controls. These results show that Cntn6 can modulate the activity of Lphn1 by direct binding and suggests that Cntn6 may prevent apoptosis thereby impinging on neurodevelopment.

## Introduction

The six members of the contactin family of immunoglobulin cell adhesion molecules (IgCAMs) play diverse roles in the nervous system (Shimoda and Watanabe, 2009; Stoeckli, 2010; Zuko et al., 2013). Contactin-1 (Cntn, a.k.a. F3) and contactin-2 (Cntn2 a.k.a. Tag-1) have been well characterized for their specialized functions in neuron–glia interaction, specifically in the paranode and juxtapararanode of the nodes of Ranvier (Peles and Salzer, 2000; Scherer and Arroyo, 2002; Poliak and Peles, 2003). The contactins act through homophilic and heterophilic interactions with various classes of proteins and form codes for specified connectivity (Stoeckli, 2010). For example, all contactin members, except Cntn6 (a.k.a. NB-3) have been proposed to participate in an IgCAM code to guide lamina-specific neurite targeting (Yamagata and Sanes, 2012). Increasing evidence suggests that Cntn6 is involved in brain development, since mice deficient for *Cntn6* display a delay in the development of the corticospinal tract, a misorientation of apical dendrites in layer V of the visual cortex, and an increase in neuronal cell death during development (Ye et al., 2008; Sakurai et al., 2009; Pinto et al., 2010; Huang et al., 2011b). A significant reduction in glutamatergic synapses was found in the hippocampus and in the cerebellum of *Cntn6* null-mutants (Sakurai et al., 2009, 2010), implicating Cntn6 in the regulation of synaptogenesis. In addition, behavioral studies have shown that *Cntn6*-deficient mice display impaired motor coordination (Takeda et al., 2003). These data indicate that Cntn6 plays a pivotal role in brain development.

A role of Cntn6 in brain development is further emphasized by genetic findings of copy number variations (CNVs) in the human *CNTN6* gene in rare cases with autism spectrum disorder (ASD) (Pinto et al., 2010; van Daalen et al., 2011; Hu et al., 2015). Furthermore, point mutations and shared CNVs between the *CNTN4* and *CNTN6* genes have also been implicated in the pathogenesis of bipolar disorder and anorexia nervosa (Pinto et al., 2010; Kerner et al., 2011; van Daalen et al., 2011; Wang et al., 2011). Finally, deletion of the tip of the short arm of chromosome 3, that harbors the *CNTN6, CNTN4* and *CHL1* genes, causes a mental retardation syndrome with ASD comorbidity, called 3p-deletion syndrome (Shuib et al., 2009). This further underscores the importance of *CNTN6* for appropriate neural development. However, it is still unknown what the molecular pathways are through which CNTN6 acts and how the loss of function of this protein contributes to disease.

The mode of action of Cntn1 and Cntn2, the best studied members of the contactin family involves the formation of multiple homo- and heterodimers in both *cis* and *trans*-configurations that are essential for the structure of the paranode and juxtaparanode (Peles et al., 1997; Rios et al., 2000; Poliak et al., 2003). Cntn4 and Cntn5 also appear to be engaged in *cis-* and *trans-*interactions with their homologs or with other proteins (Traka et al., 2003; Cui et al., 2004; Osterfield et al., 2008; Ye et al., 2008, 2011; Bouyain and Watkins, 2010; Shimoda et al., 2012; Yamagata and Sanes, 2012; Ashrafi et al., 2014; Osterhout et al., 2015). Cntn6 is known to complex with other membrane proteins as well, including Ptpra, Ptprg, PTPσ, Notch, and Chl1 (Cui et al., 2004; Hu et al., 2006; Ye et al., 2008; Bouyain and Watkins, 2010; Zuko et al., 2011; Huang et al., 2016) Thus, interaction with other protein partners appears as a common theme in the mode of action of contactins. Therefore, we further examined protein networks in which Cntn6 participates. We demonstrate here that Cntn6 binds to the cell adhesion G-protein-coupled receptor (adhesion GPCR) latrophilin-1 (Lphn1, ADGRL1, a.k.a. CIRL1/CL) forming a silenced *cis*-complex. Loss of Cntn6 results in inhibition of neurite outgrowth and an increased neuronal cell death due to unoccupied Lphn1. This study indicates that Cntn6 serves as an endogenous ligand for Lphn1 thereby controlling apoptosis. This conclusion was supported by *in vivo* analyses of the *Cntn6*^-/-^** mouse brain displaying increased apoptosis which links Cntn6 to one of the pathogenic pathways of autism (Wei et al., 2014).

## Materials and Methods

### Animals and Tissue Treatment

B57BL/6 and *Cntn6^-/-^* mice were obtained from Charles River and Nagaoka University (Takeda et al., 2003), respectively. Mice were maintained on a 12-h light/dark cycle with *ad libitum* food and water in an animal facility at Brain Center Rudolf Magnus, Utrecht University. For immunohistochemistry, P14 mouse pups were anesthetized with an overdose of sodium pentobarbital (19.4 μl/gr) and were perfused intracardially with 0.9% saline, followed by 4% PFA in PBS, pH 7.5. Brains were post fixed in 4% PFA before transferred to 30% sucrose for cryopreservation. Tissue was sectioned at 40 μm sections and free-floating sections were stored in PBS with 0.02% sodium azide until immunohistochemistry was performed. For *in situ* hybridization, P7 mouse pups were killed by decapitation and their brains were quickly dissected and flash-frozen in 2-methylbutane. Brains were sliced into 16 μm sections using a cryostat and mounted onto Superfrost slides (VWR).

### Cell Adhesion Assay

Cell adhesion assays were performed with HEK293 cells as previously described Ko et al. (2009). HEK293 cells were cotransfected either with pCMV-EGFP-N1 or pCAG-DsRed and full-length pcDNA3.1-Cntn6, pcDNA3.1-Lphn1, pCAG-HA-Nlgn1 and pCAG-HA-Nrxn1β^-^ (latter two were gifts from Dr. Scheiffele) expression constructs. After 48 h, the cells were detached using 1 mM EDTA in PBS, pH 7.4, and centrifuged at 1000 rpm for 5 min. The pellets were resuspended in suspension medium (10% HIFCS, 50 mM Hepes-NaOH, pH 7.4, 10 mM CaCl_2_ and 10 mM MgCl_2_) and combined to a total of 5x10^6^ (1:1) in 0.3 ml total volume of 0.5 ml eppendorf tubes. The cell mixtures were incubated at RT under gentle agitation. The extent of cell aggregation was assessed at 90 min by removing aliquots, spotting them onto culture slides (BD Falcon), and imaging by a Zeiss Axiosop A1 microscope. The resulting images were then analyzed by counting the number and size of particles using ImageJ. An arbitrary value for particle size was then set as a threshold based on negative control values. The aggregation index was calculated by expressing the number of particles participating in aggregation as a percentage of the total particles in 10 to 5 fields of 1.509 mm^2^ per cell suspension combination of each independent experiment (*n* = 3). Statistical analysis was carried out using unpaired Student’s *t-*test.

### Cell Culture and Transfection

HEK293 cells were maintained in high glucose Dulbecco’s modified Eagle’s medium 5 g/L glucose (DMEM; Gibco). Cell culture media were supplemented with 10% (v/v) heat-inactivated fetal bovine serum (FBS, Lonza, BioWhittaker), 2 mM L-glutamine (PAA) and 1x penicillin/streptomycin (pen/strep; PAA) and cultured in a humidified atmosphere with 5% CO_2_ at 37°C. HEK293 cells were transfected using polyethylenimine (PEI; Polysciences) (Reed et al., 2006) or Lipofectamine LTX (Invitrogen, according to manufacturers manual). For the examination of Cntn6 effects on Lphn1 autoproteolysis, pcDNA3.1-Cntn6 and pcDNA3.1-HA-Lphn1 were cotransfected in HEK293 cells in the ratios 6:3 and 6:1 respectively. The transfected cells were lysed and analyzed on Western blot.

### Cell Surface Binding Assay

To investigate whether Cntn6 interacts with Lphn1, a cell surface binding assay was used with slight modifications (Shimoda et al., 2012). Transfection of HEK293 cells with pIGplus-RGMa-Fc or pCr/TEV-ectoLphn1-Fc was performed. Forty eight hours after transfection the medium with soluble RGMa-Fc or Lphn1-Fc was concentrated through a 50,000 kDa column (YM-50, Milipore). The concentrated proteins were supplemented with Dulbecco’s modified Eagle’s medium 1 g/L glucose (DMEM; Gibco, Invitrogen) with 2 mM L-glutamine (PAA) and 1x penicillin/streptomycin (pen/strep; PAA) and distributed in 6-well plates with HEK293 cells transfected with pcDNA3.1-neogenin-myc or pcDNA3.1-HA-Cntn6 constructs. Binding between the proteins was allowed overnight in a humidified atmosphere with 5% CO_2_ at 37°C. Cells were fixed with 4% PFA in PBS, pH 7.4, and 0.01% sodium azide until immunocytochemistry was performed. For the cell surface binding analysis, images from the Zeiss Axioscop A1 were used. Analyses were performed of about 400 transfected cells per condition of each independent experiment (*n* = 3). The images were analyzed by quantification of the number of double labeled cells as a percentage of the total amount of transfected cells in ImageJ. Statistical analysis was carried out using unpaired Student’s *t*.

### Construction of Expression Vectors

A biotin- and GFP-tagged extracellular rat Cntn6 (Cntn6-TMGFPBio) fusion protein was generated by subcloning the coding sequence of the extracellular Cntn6 domains (NM_013225.1: nt 248–3169), excluding the coding sequence of the GPI anchor. This was amplified from wild-type Cntn6 cDNA (pcDNA3.1-Cntn6) and ligated to the sequence of plexin-A1 transmembrane domain coding sequence (NM_008881.2: nt 3962–4123). The coding sequences of a five glycine linker and intracellular GFP and biotin tags followed and were inserted in a pcDNA3.1(-)/myc-His (Invitrogen) vector backbone. The control vector (TMGFPBio) is identical but it is truncated beyond the transmembrane domain.

### Construction of Lphn1 shRNA Vectors

The pSUPER vector backbone (OligoEngine) was used to synthesize short hairpin RNA (shRNA) designed against Lphn1. This vector backbone carries the polymerase-III H1-RNA promotor, which produces small RNA transcripts lacking a polyadenosine tail and has a well-defined start of transcription and termination signal (Brummelkamp et al., 2002a,b). The following sequences were used: Lphn1 shRNA1: GCAACACCATCCACAAGAA, Lphn1 shRNA2: CAAGGGAACTCGAGGAATT, Lphn1 shRNA3: TCTCAGAGCTGGTGCACAA, Lphn1 shRNA4: GGGCAAATGCAGTTGGTCA. A non-targeting shRNA with a fully scrambled targeting sequence was designed as a control with the following sequence: GCTCTTAATCGCAAATACA. To examine the efficiency of Lphn1 knockdown, HEK293 cells were cotransfected with pcDNA3.1-HA-Lphn1-GFP and the Lphn1 shRNA constructs in a 1:2 ratio. The Lphn1 knockdown was examined in one experiment (*n* = 1) 3 days after transfection by quantification of fluorescence of about 1500 cells from five fields of 0.4 mm^2^ per cell suspension of each transfection condition. The lysates were used in Western blot experiments and blot quantification was done by ImageJ.

### Ethics Statement

The experiments performed in this study were approved by the Experimental Animal Committee (DEC) of Utrecht (2010.I.06.073). All animal experiments were conducted in agreement with Dutch law (Wet op de Dierproeven, 1996) and European regulations (Guideline 86/609/EEC) related to the protection of vertebrate animals used for experimental and other scientific purposes.

### Immunostaining

Immunocytochemistry was performed after HEK293 cells were fixed with 4% PFA for 15 min at room temperature (RT) and washed in PBS (pH 7.4). The HEK293 cells were incubated in goat blocking buffer [PBS, 1% bovine serum albumin (BSA), 2% normal goat serum, 0.3% Triton X-100] for 1 h at RT. HEK293 cells were incubated with primary antibodies in goat blocking buffer overnight at 4°C. Cells were washed in PBS and incubated with species-specific secondary antibodies conjugated to Alexa Fluor (Invitrogen) 1:2000 for 1 h at RT. Cells were washed in PBS and incubated with 4′,6-diamidino-2-phenylindole (DAPI) (Sigma) before embedding. Images were captured by epifluorescence illumination on a Zeiss Axioscop A1. The primary antibodies that were used: rabbit anti-Cntn6-45 (antiserum produced by Harlan) 1:1000; rat anti-GFP (Chromotek) 1:500; rabbit anti-myc (Abcam) 1:500; and mouse anti-Fc-HRP (Bioconnect) 1:2500. Immunocytochemistry on primary cultures was performed as described before, with the following primary antibodies: rat anti-GFP (Chromotek) 1:500; rabbit anti-Caspase-3 (Cell Signaling) 1:1000; rabbit anti-Flag (Sigma) 1:250; rat anti-HA (Roche) 1:500; sheep anti-Cntn6 (R&D systems) 1:100; rabbit anti-Lphn1-p85 1:1000. Images were captured by confocal laser scanning microscopy (Olympus FV1000) by a Zeiss Axiosop A1.

For immunohistochemistry, the visual cortex was identified using standard stereotaxic coordinates (-2.80 mm to bregma). The sections were washed with PBS and incubated for 45 min in blocking buffer [1% BSA, 0.2% fish skin gelatin (Sigma), 0.1% Triton X-100 in PBS] and washed again. Sections incubated for 10 min in permeabilization buffer (0.3% Triton X-100 in PBS) before 2 h incubation with primary antibody in blocking buffer at 4°C. The sections were washed in PBS and pre-incubated with blocking buffer before incubating with species-specific secondary antibodies conjugated to Alexa Fluor (Invitrogen) 1:500 for 2 h at RT. A 10 min DAPI incubation was performed after the sections were washed in PBS. The sections were embedded with Polyvinyl alcohol mounting medium with DABCO antifading (Fluka) onto glass slides after additional PBS wash steps. Primary antibodies that were used: sheep anti-Cntn6 (R&D systems) 1: 100; chick anti-Lphn1-p85 1: 500; rabbit anti-Caspase-3 (Cell Signaling) 1:400. Images were captured by confocal laser scanning microscopy (Olympus FV1000). Quantifications of caspase-3 immunoreactivity in the visual cortex were performed under a Zeiss Axioscop A1. At least three sections were analyzed from of *Cntn6^+/+^* and *Cntn6^-/-^* P14 animals (*n* = 5). Statistical analysis was carried out using unpaired Student’s *t*.

### Immunoprecipitation

Immunoprecipitation (IP) experiments were performed using GFP-Trap-A agarose beads (Chromotek, according to manufacturers manual). For proteomics, HEK293 cells expressing the indicated GFP-tagged fusion proteins were collected in ice-cold PBS and centrifuged at 1000 rpm in a precooled centrifuge at 4°C for 5 min. Cell pellets were lysed in lysis buffer [10 mM Tris-HCl, pH 7.5, 150 mM NaCl, 0.5 mM EDTA, 0.5% NP40, 1 mM PMSF and Complete protease inhibitor cocktail (Roche)], incubated on ice for 30 min and centrifuged at 13,200 rpm at 4°C for 10 min. Cleared supernatant containing roughly 5.4 – 6.6 mg of protein was mixed with 50 μl GFP-Trap-A agarose beads (Chromotek), which had been equilibrated in dilution buffer [10 mM Tris-HCl, pH 7.5, 150 mM NaCl, 0.5 mM EDTA, 1 mM PMSF and Complete protease inhibitor cocktail (Roche)] at 4°C. After 1.5 h incubation at 4°C, beads were washed two times in dilution buffer. Precipitated proteins were eluted by boiling the pull-down samples in NuPAGE LDS sample buffer (Invitrogen) containing 2% β-mercaptoethanol at 95°C for 10 min.

For *in vitro* coIP, HEK293 cells were cotransfected with the following constructs: pcDNA3.1-Cntn6-TMGFPBio, pcDNA3.1-Lphn1-GFP or pCMV-EGFP-N1 with either pcDNA3.1-HA-Lphn1 or pcDNA3.1-HA-Cntn6. The pull-down experiments were performed using 1.8 – 2.2 mg total protein and 25 μl GFP-Trap-A agarose beads, as previously described. For endogenous coIP, P14 mouse cortex was lysed in lysis/washing buffer [20 mM Tris-HCl, pH 7.5, 150 mM NaCl, 1 mM EDTA, 5% Glycerol, 1% CHAPS, 1 mM PMSF, Complete protease inhibitor cocktail (Roche) and phosphatase inhibitor cocktail (Sigma)], incubated for 30 min on ice and centrifuged at 13,200 rpm at 4°C for 10 min. Cleared supernatants containing roughly 4 mg of protein were incubated at RT for 30 min with 50 μl paramagnetic beads with coupled recombinant protein G (Dynabeads Protein-G, Invitrogen), which were pre-incubated with 10 μg sheep anti-Cntn6 or normal sheep IgG (Milipore) antibodies in PBS and 0.02% Tween. Pull-down samples were washed three times in lysis/washing buffer and precipitated proteins were eluted by boiling in NuPAGE LDS sample buffer containing 2% β-mercaptoethanol at 70°C for 10 min or by 5 min incubation with elution buffer (0.1 M Glycine, pH 2.5) before adding 5 μl Tris-HCl (1 M, pH 8.5).

### *In situ* Hybridization

Non-radioactive *in situ* hybridization was performed as previously described Pasterkamp et al. (1998). In brief, probe sequences for Cntn6 (NM_017383.3: nt 283–876) or Lphn1 (NM_181039.2: nt 5203–5585) were polymerase chain reaction (PCR)-amplified from cDNA. Digoxigenin (DIG)-labeled RNA probes were generated by a RNA polymerase reaction using 10x DIG RNA labeling mix (ENZO). Tissue sections were post-fixed in 4% PFA in PBS, pH 7.40 for 20 min at RT. To enhance tissue penetration and decrease a specific background staining, sections were acetylated with 0.25% acetic anhydride in 0.1 M triethanolamine and 0.06% HCl for 10 min at RT. Sections were prehybridized for 2 hrs at RT in hybridization buffer (50% formamide, 5x Denhardt’s solution, 5x SSC, 250 μg/ml baker’s yeast tRNA and 500 μg/ml sonicated salmon sperm DNA). Hybridization was performed for 15 h at 68°C, using 400 ng/ml denatured DIG-labeled probe diluted in hybridization buffer. After hybridization, sections were first washed briefly in 2x SSC followed by incubation in 0.2x SCC for 2 hrs at 68°C. Sections were adjusted to RT in 0.2x SSC for 5 min. DIG-labeled RNA hybrids were detected with anti-DIG Fab fragments conjugated to AP (Boehringer) diluted in 1:2500 in TBS (pH 7.4) overnight at 4°C. Binding of AP-labeled antibody was visualized by incubating the sections in detection buffer (100 mM Tris-HCl, pH 9.5, 100 mM NaCl and 50 mM MgCl_2_) containing 240 μg/ml levamisole and nitroblue tetrazolium chloride/5-bromo-4-chloro-3-indolyl-phosphatase (NBT/BCIP, Roche) for 14 h at RT. Sections subjected to the entire *in situ* hybridization procedure, but with no probe or sense probe added, did not exhibit specific hybridization signals. The specificity of the *in situ* hybridization procedure was also inferred from the clearly distinct gene expression patterns observed. Staining was visualized using a Zeiss Axioskop 2 microscope.

### Mass Spectrometry: RP-NanoLC-MS/MS

The data was acquired using an LTQ-Orbitrap coupled to an Agilent 1200 system or an Orbitrap Q Exactive mass spectrometer connected to an Agilent 1290 system. In case of the LTQ-Orbitrap, peptides were first trapped ((Dr Maisch GmbH) Reprosil C18, 3 μm, 2 cm × 100 μm) before being separated on an analytical column (50 μm × 400 mm, 3 μm, 120 Å Reprosil C18-AQ). Trapping was performed at 5 μl/min for 10 min in solvent A (0.1 M acetic acid in water), and the gradient was as follows; 10 – 37% solvent B (0.1 M acetic acid in 80% acetonitrile) in 30 min, 37–100% B in 2 min, 100% B for 3 min, and finally solvent A for 15 min. Flow was passively split to 100 nl min^-1^. Data was acquired in a data-dependent manner, to automatically switch between MS and MS/MS. Full scan MS spectra from m/z 350 to 1500 were acquired in the Orbitrap at a target value of 5e5 with a resolution of 60,000 at m/z 400 in case of the LTQ-Orbitrap XL and 30,000 for the LTQ-Orbitrap Discovery. The five most intense ions were selected for fragmentation in the linear ion trap at normalized collision energy of 35% after the accumulation of a target value of 10,000. In case of the Q Exactive samples were first trapped [(Dr Maisch GmbH) Reprosil C18, 3 μm, 2 cm × 100 μm) before being separated on an analytical column (Agilent Poroshell EC-C18, 2.7 μm, 40 cm × 50 μm)]. Trapping was performed for 10 min in solvent A and the gradient was as follows; 13–41% solvent B in 35 min, 41–100% in 3 min and finally solvent A for 10 min. Flow was passively split to 100 nl min^-1^. The mass spectrometer was operated in data-dependent mode. Full scan MS spectra from *m/z* 350 – 1500 were acquired at a resolution of 35,000 at *m/z* 400 after accumulation to a target value of 3e6. Up to ten most intense precursor ions were selected for fragmentation. HCD fragmentation was performed at normalized collision energy of 25% after the accumulation to a target value of 5e4. MS/MS was acquired at a resolution of 17.500. In all cases nano-electrospray was performed at 1.7 kV using an in-house made gold-coated fused silica capillary (o.d. 360 μm; i.d. 20 μm; tip i.d. 10 μm).

### Neuronal Culture

P0–P1 mouse cerebral cortices were dissected and washed three times in L15 medium (Gibco) with 7 mM HEPES (L15-HEPES) and once in L15-HEPES with 0.5 M EDTA. For dissociation, the tissue was incubated in 0.25% trypsin (PAA) in L15-HEPES for 20 min at 37°C, followed by trituration in complete Neurobasal [Neurobasal medium supplemented with 2% B27 (Invitrogen), 25 μM β-mercaptoethanol, 0.5 mM L-glutamine (PAA) and 1x penicillin/streptomycin (pen/strep; PAA)] with 20 μg/ml DNase I (Roche) using a fire-polished Pasteur pipette. Dissociated cortical neurons were run through a 100 μm cell strainer (BD Falcon) and plated in complete Neurobasal medium at 150 K/well of 12-well plates onto PDL (20 μg/ml) and laminin (40 μg/ml), Cntn6.6His (R&D Systems, 10 μg/ml) or BSA (Sigma–Aldrich, 10 μg/ml) coated glass coverslips.

### Neuronal Transfection and Analysis

At DIV2, neurons in culture were cotransfected by Lipofectamine LTX (Invitrogen, according to manufacturers protocol), with pCAG-aGFP and either full-length pcDNA3.1-Cntn6, pcDNA3.1-Lphn1, combination of both or empty pcDNA3.1 control vector. For colocalization experiments, neurons were cotransfected with pcDNA3.1-HA-Lphn1, pCMV-Flag-Cntn6, or a combination of both. For Lphn1 knockdown experiments, neurons were cotransfected with pCAG-aGFP and a pSUPER vector carrying a scrambled sequence or a sequence designed against Lphn1 (the aforementioned shRNA3 and shRNA4) in a 1:12.5 ratio.

Neuronal medium was replaced with complete Neurobasal without antibiotics. A total of 0.5 μg DNA was incubated with 1.68 μl PLUS reagent in 200 μl Optimem (Invitrogen) for 10 min. One microliter Lipofectamine LTX was added to the DNA mix and was incubated for 30 min before addition to the neurons. At DIV5, neurons were washed with PBS, fixed with 4% PFA and 4% Sucrose in PBS, pH 7.4 for 20 min at 37°C before washing 3 more times with PBS. After immunostaining, images from the Zeiss Axioscop A1 were taken. For analysis of neuronal morphological parameters, about 110 transfected neurons were examined per condition of each independent experiment (*n* = 3). WIS-Neuromath (Weizmann Institute) software was used for determining morphological parameters (Rishal et al., 2013), which included total branch number, soma size, total outgrowth and maximal process length. For analysis of neuronal apoptosis in the protein overexpression experiments, the immunoreactivity of caspase-3 in about 60 transfected neurons was quantified per condition of each independent experiment (*n* = 3). For analysis of neuronal apoptosis in the Lphn1 knockdown experiments, the immunoreactivity of caspase-3 in about 60 transfected neurons was quantified per condition of each independent experiment (*n* = 5 for *Cntn6^+/+^* and *n* = 4 for *Cntn6^-/-^* cultures). Positive neurons were analyzed by quantification of the number of double-labeled cells as a percentage of the total amount of transfected cells in ImageJ. Statistical analyses were carried out using unpaired Student’s *t*.

### Protein Separation and Digestion

Thirty microliter of each sample ran on a 12% Bis-Tris 1D SDS-PAGE gel (Biorad) either for 2–3 cm or ran completely and stained with colloidal coomassie dye G-250 (Gel Code Blue Stain Reagent, Thermo Scientific). The lane was cut into bands, which were treated with 6.5 mM dithiothreitol (DTT) for 1 h at 60°C for reduction and 54 mM iodoacetamide for 30 min for alkylation. The proteins were digested overnight with trypsin (Promega) at 37°C. The peptides were extracted with acetonitrile (ACN) and dried in a vacuum concentrator.

### Proteomics Data Analysis

Raw files were processed using Proteome Discoverer 1.3 (version 1.3.0.339, Thermo Scientific, Bremen, Germany). The database search was performed against the Swissprot database (version August 2014) using Mascot (version 2.4.1, Matrix Science, UK) as search engine. Carbamidomethylation of cysteines was set as a fixed modification and oxidation of methionine was set as a variable modification. Trypsin was specified as enzyme and up to two miss cleavages were allowed. Data filtering was performed using percolator, resulting in 1% false discovery rate (FDR). Additional filter was Mascot ion score >20. Raw files corresponding to one sample were merged into one result file.

Data was further analyzed with Saint (Choi et al., 2011) using the Crapome web interface^1^ in order to identify interacting proteins. Default settings were used for calculating the FC-A and FC-B score. The probability score was calculated using Saint Express performing 20,000 iterations.

### PSD Preparation

To isolate postsynaptic densities (PSDs) from rat cortex or hippocampus, a modification of the method of Gardoni et al. (1998) was used. In brief, whole brain from 1 adult rat rapidly dissected and frozen on dry ice within 2 min to avoid postmortem intracellular protein trafficking (Suzuki et al., 1994). Homogenization was carried out by 20 strokes in a Teflon—glass homogenizer (700 rpm) in 10 ml/g of cold 0.32 M sucrose containing 1 mM HEPES, 1 mM MgCl2, 1 mM NaHCO2, and 0.1 mM phenylmethylsulfonyl fluoride (PMSF) (pH 7.4) in the presence of a complete set of protease and phosphatase inhibitors (Sigma). The homogenized tissue (H) was centrifuged at 1,000 *g* for 10 min at 4°C in a Sorvall centrifuge with SM24 inner rotor. The resulting supernatant was centrifuged at 13,000 *g* for 15 min at 4°C with the Sorvall centrifuge to obtain the crude membrane fraction (P2). The pellet was resuspended in 5 ml/g of 0.32 M sucrose containing 1 mM HEPES, 1 mM NaHCO2, and 0.1 mM PMSF (pH 7.4), overlaid on a sucrose gradient (0.85—1.0—1.2 M), and centrifuged at 82.500 *g* for 2 h at 4°C in a Beckman ultracentrifuge with SW41 swingout rotors. The synaptosome fraction (F1) between 1.0 M and 1.2 M sucrose was removed and diluted in 75 ml total volume in 0.5% Triton X-100, 0.32 M sucrose, 1 mM HEPES. This solution was spun down at 82,500 *g* for 30 min at 4°C in the Beckman ultracentrifuge. The pellet was collected, resuspended and pottered by 20 strokes in a Teflon-glass homogenizer in 3 ml 0.32 M sucrose, 1 mM HEPES. The Triton insoluble postsynaptic fraction (P3) was removed and stored while the rest was layered on a sucrose gradient (1.0—1.5—2.1 M), and centrifuged at 100,000 *g* at 4°C for 2 h the Beckman ultracentrifuge. The fraction between 1.5 M and 2.1 M was removed and diluted in total volume of 13 ml with 0.5% Triton X-100 and 75 mM KCl. The enriched PSD fraction (P4) was finally collected by centrifugation at 100,000 *g* at 4°C for 30 min by the Beckman ultracentrifuge, and stored at -80°C.

### Real-Time PCR

Mouse brain RNA was isolated from wild-type embryos at developmental stages E12.5, E14.5, E16.5, E18.5 and postnatal stages P7 and adult. To determine levels of *Cntn6* expression, one-step qPCR was performed using a Quantifast SYBR Green RT PCR kit (Qiagen) and a LightCycler (Roche, Mannheim, Germany), according to manufacturers instruction. GAPDH primers: FW: CATCAAGAAGGTGGTGAAGC, RT: ACCACCCTGTTGCTGTAG, Cntn6 primers: FW: CCCAAGTTCCAACAAGAGGA, RV: GCCACGTGTACGAAGGATT.

### Western Blotting

Cells were collected with a cell scraper in ice-cold PBS (pH 7.4) and centrifuged at 1000 rpm for 5 min in a precooled centrifuge at 4°C. The cell pellet was resuspended in ice-cold lysis buffer (20 mM Tris-HCl, pH 8, 150 mM KCl, 1% Triton X-100, 1 mM PMSF and Complete protease inhibitor cocktail (Roche)), incubated on ice for 10 min, followed by centrifugation at 13,200 rpm for 10 min at 4°C. The supernatant was collected, NuPAGE LDS sample buffer (Invitrogen) containing 2% β-mercaptoethanol was added and samples were boiled for 5 min at 90°C. Proteins were separated in 8% SDS-PAGE gels and transferred onto nitrocellulose membrane (Hybond-C Extra; Amersham). Membranes were incubated in blocking buffer [PBS, 0.05% (v/v) Tween 20 and 5% milk powder] for 30 min at RT. Membranes were incubated with corresponding primary antibodies in blocking buffer overnight at 4°C. Antibodies used: rat anti-GFP (Chromotek) 1:1000; rat anti-HA (Roche) 1:100, rabbit anti-Cntn6-45 (Harlan) 1:2000; chick anti-Lphn1-p120 and rabbit anti-Lphn1-p85 1:2500, rabbit anti-SynapsinI (Sigma) 1:2000, mouse anti-PSD95 (Milipore) 1:250, mouse anti-βActin (Sigma) 1:1000. Blots were incubated with SuperSignal West Dura Extended Duration Substrate (Pierce) and exposed to ECL films (Pierce) or imaged by FluorchemE Digital Darkroom (Cell Biosciences). ImageJ was used for blot quantification.

## Results

### Identification of Lphn1 As Interacting Partner of Cntn6

**FIGURE 1:**
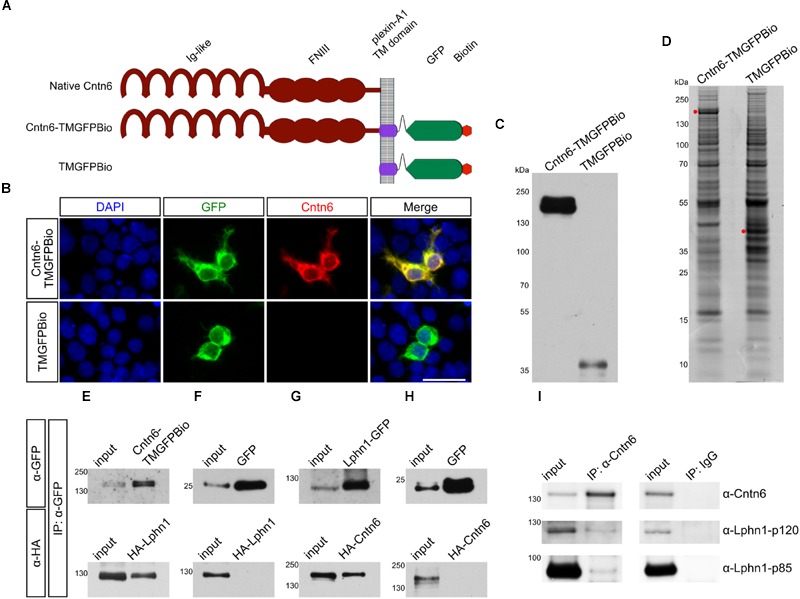
**Interaction of Cntn6 and Lphn1 *in vitro* and *in vivo*. (A)** The architecture of the native Cntn6 protein and the structures of the Cntn6 and control fusion proteins tagged with GFP and biotin. **(B)** Expression of Cntn6-TMGFPBio and TMGFPBio in HEK293 cells, detected by fluorescence (green), anti-Cntn6 antibody (red), and DAPI (blue) staining. Scale bar represents 30 μm. **(C)** Precipitations were performed by anti-GFP-coupled beads and eluates from anti-GFP-coupled beads were analyzed on Western blot using an anti-GFP antibody. **(D)** Coomassie blue stained the NuPage 4–12% gels, which were submitted to mass spectrometry analysis. Red dots indicate respective expressed fusion proteins. **(E–H)** HEK293 cells were cotransfected with Cntn6 and Lphn1 expression plasmids tagged with either GFP or HA. After IP of the GFP-tagged protein by anti-GFP antibodies the eluates were analyzed by Western blot. **(E)** Immunoblotting with anti-HA and anti-GFP antibodies revealed HA-Lphn1 coprecipitation with Cntn6-TMGFPBio, **(F)** but not with control GFP. **(G)** HA-Cntn6 was coprecipitated with Lphn1-GFP, **(H)** but not with control GFP. **(I)** Proteins were IPed from wild-type P14 mouse cortex lysates using an anti-Cntn6 antibody. Blots stained with antibodies against Cntn6 and both p85- and p120-fragments of Lphn1 revealed interaction between Cntn6 and Lphn1. No coprecipitation was found in normal IgG control IP. Molecular weights are as follows: Cntn6-TMGFPBio = 147.8 kDa; TMGFPBio = 39.6 kDA; Lphn1-GFP = 125 kDa; GFP = 27 kDa; HA-Cntn6 = 141 kDa; HA-Lphn1 = 131 kDa; Cntn6 = 130 kDa; Lphn1-p85 = 85 kDa; Lphn1-p120 = 120 kDa. Ig-like, immunoglobulin-like; FNIII, fibronectin type III; TM, transmembrane domain; GFP, green fluorescent protein; Bio, biotin.

An unbiased proteomics approach using Cntn6 protein fused to the Plexin A2 transmembrane domain and GFP (Cntn6-TMGFPBio and control TMGFPBio) was used to analyze immunoprecipitates (IP) of this protein expressed in HEK293 cells (**Figures 1A,B**). These cells are known to express a large range of proteins (Geiger et al., 2012). Following IP experiments, Cntn6 fusion and control proteins were detected at 148 and 40 kDa, respectively, by Western blotting and Coomassie blue staining (**Figures 1C,D**). Raw mass spectrometry data was analyzed with the Mascot search engine and scores were assigned to identify peptides. In comparison to control experiments, confidence scores using Saint scoring (Choi et al., 2011) were assigned to the identified proteins. A ranked list of putative interacting proteins was obtained representing proteins that were significantly higher or exclusively present in the Cntn6 fusion protein pull-down samples (**Table 1**). The highest scoring transmembrane protein was the adhesion G protein-coupled receptor Lphn1. We therefore further examined the potential of Cntn6 to interact and function with Lphn1.

**Table 1:** Top Cntn6 interacting proteins.

PROTID	Gene	FC-A	FC-B	SAINT	Protein	Cellular localization
P97528	*Cntn6*	44.23	44.11	1	Contactin-6	Cell membrane
O94910	*LPHN1*	16.87	13.16	0.88	Latrophilin-1	Cell membrane; Synapse
Q9UBS4	*DNAJB11*	5.66	5.6	0.84	DnaJ homolog subfamily B member 11	Endoplasmic reticulum
O94779	*CNTN5*	11.64	5.71	0.67	Contactin-5	Cell membrane
P27797	*CALR*	5.47	3.48	0.65	Calreticulin	Endoplasmic reticulum; Cytoplasm, Extracellular space

*The proteins that were coimmunoprecipitated with Cntn6-TMGFPBio and detected by mass spectrometry are listed according their SAINT score. Data from triplicate IPs were compared to control using CRAPome, which employs SAINT and FC statistical analyses. This table lists the protein identification code (PROTID) and its related gene name, together with FC-A, FC-B, and SAINT scoring. The standard CRAPome Fold Change calculations (FC-A) estimates the background by averaging the spectral counts across the selected controls. Another more stringent Fold Change (FC-B) calculation estimates the background by combining the top 3 values for each identified interacting protein, while SAINT reports a probability of true interaction. Cellular localization was determined by searching Uniprot (UniProt Consortium, 2015). See the Supplementary Proteomics Datasheet for the complete raw data.*

### Cntn6 Interacts With Lphn1 *In vitro* and *In vivo*

To validate the association of Cntn6 and Lphn1, HEK293 cells were cotransfected with Cntn6-TMGFPBio and HA-Lphn1 expression plasmids, and appropriate controls. IP analysis by Western blotting demonstrated coprecipitation of HA-Lphn1 with Cntn6-TMGFPBio, but not with controls GFP (**Figures 1E,F**). Similarly, HA-Cntn6 and Lphn1-GFP coprecipitated in cotransfected HEK293 cells (**Figure 1G**). HEK293 cells coexpressing GFP and HA-Cntn6 were used as a control experiment, and IP did not result in HA-Cntn6 coprecipitation with control GFP (**Figure 1H**).

Next we investigated the association of endogenous Cntn6 and Lphn1 in P14 wild-type mouse cerebral cortex. These experiments resulted in the coprecipitation of both the p85- and p120-fragments of Lphn1 with Cntn6 indicating that an endogenous interaction of Lphn1 and Cntn6 exists in the brain (**Figure 1I**).

### Cntn6 and Lphn1 Interact in a *Cis*-Complex

**FIGURE 2:**
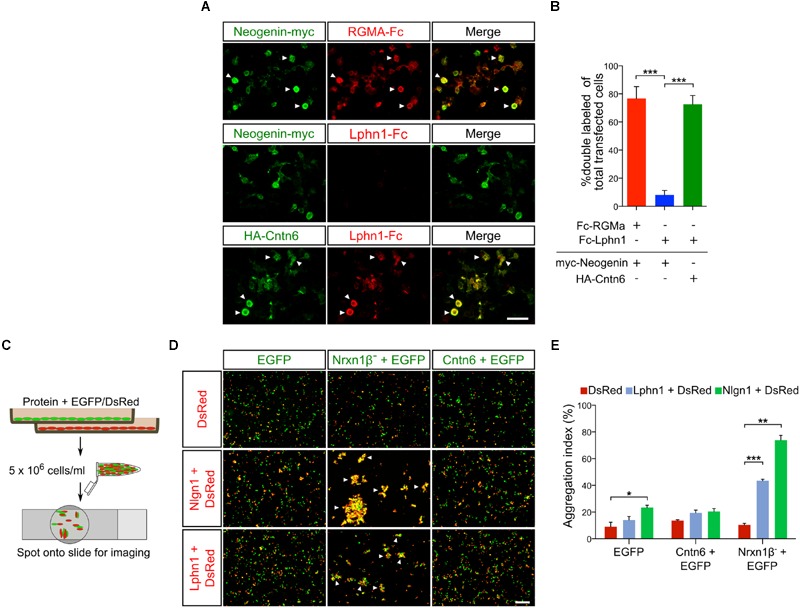
**Cntn6 and Lphn1 interact in *cis*-configuration. (A)** HEK293 cells expressing myc-neogenin or HA-Cntn6 were incubated with soluble ecto-domains of RGMA-Fc and Lphn1-Fc. Upper panel: neogenin-myc-expressing cells (green) bound soluble RGMA-Fc (red), but not Lphn1-Fc (red) (middle panel). Lower panel: HA-Cntn6-expressing cells (green) bound soluble Lphn1-Fc (red). Scale bar represents 50 μm. Arrowheads indicate green and red overlay. **(B)** Quantification of about 400 transfected cells per transfection condition of each independent cell surface binding assay (*n* = 3) was performed. **(C)** Schematic overview of the intercellular cell adhesion assay, in which populations of HEK293 cells were cotransfected with different cell adhesion proteins and either EGFP or DsRed expression plasmids. Two HEK293 cells populations were combined in a total of 5 × 10^6^ cells/ml. After incubation, cell suspensions were spotted onto slides for imaging by fluorescence microscopy. **(D)** Cells expressing either EGFP alone or together with Nrxn1β^-^ or Cntn6 (green) were mixed with cells expressing DsRed alone or together with Nlgn1 or Lphn1 (red). Aggregation of cells expressing Nrxn1β^-^ + EGFP with Nlgn1 + DsRed and Lphn1 + DsRed was observed. There was no aggregation of cells expressing Cntn6 + EGFP with Lphn1 + DsRed. The scale bar represents 200 μm. Arrowheads indicate cell aggregates. **(E)** Aggregation index was determined from five fields of 1.509 mm^2^ per cell suspension combination of each independent cell adhesion assay (*n* = 3). Analysis was performed using unpaired Student’s *t* test. The graph bars are presented as mean ± SEM. ^∗^*p* < 0.05, ^∗∗^*p* < 0.01, ^∗∗∗^*p* < 0.001.

The configuration of the Cntn6-Lphn1 interaction was determined through cell assays. Firstly, cell surface binding assays confirmed the biochemical interaction between Lphn1 and Cntn6: the soluble, tagged ectodomain Lphn1-Fc bound to membrane-bound HA-Cntn6 (**Figures 2A,B**). The well-characterized *trans*-interacting proteins neogenin and RGMa were used as positive controls in this assay (Yamashita et al., 2007; Itokazu et al., 2012). Secondly, to study whether Cntn6 and Lphn1 interacted in *cis* or *trans*, cell adhesion assays were performed involving separate populations of HEK293 cells cotransfected with either native Cntn6 and EGFP or with Lphn1 and DsRed expression plasmids (**Figures 2C,D**). As a positive control, cells were cotransfected with neuroligin-1 (Nlgn1) and DsRed or with neurexin-1β^-^ (Nrxn1β^-^) and EGFP expression plasmids (Ichtchenko et al., 1995). As negative controls, cells were transfected with either DsRed or EGFP expression plasmids only. Since Nlgn1 and Lphn1 both individually interact with Nrxn1β^-^ (Nguyen and Südhof, 1997; Boucard et al., 2005, 2012) these proteins were used as positive controls. Indeed, a significant increase in the number of adhering cell clumps was observed when Nrxn1β^-^-expressing cells were mixed and incubated either with Nlgn1- or Lphn1-expressing cells (**Figures 2D,E**), demonstrating the validity of the assay. A significantly smaller degree of cell-aggregation was observed in the mixture of Nlgn1- (red) with EGFP-expressing (green) cells (**Figure 2C**). However, these aggregates comprised only red cells, which indicated Nlgn1’s capability to homodimerize in *trans*. No cell-aggregation was found when Cntn6-expressing cells were mixed with Lphn1-expressing cells. These data show that the binding of Lphn1 to Cntn6 cannot occur when the proteins are expressed on opposing cells in *trans*-configuration. Together with the results of the cell surface binding experiments and coIP, the data indicate that Cntn6 and Lphn1 bind each other in *cis-*configuration and may form a heterodimer.

In view of the Cntn6-Lphn1 *cis*-interaction and the proposed localization of Lphn1 and Cntn6 in the synapse (Sakurai et al., 2009; Silva et al., 2011), we examined the distribution of Lphn1 and Cntn6 across different synaptic fractions. Both proteins were present in the postsynaptic density fractions (Supplementary Figure 1). This supports the conclusion that Cntn6 and Lphn1 interact in *cis* and suggests that they form a postsynaptic complex.

### Coexpression of Cntn6 and Lphn1 in the Mouse Brain

**FIGURE 3:**
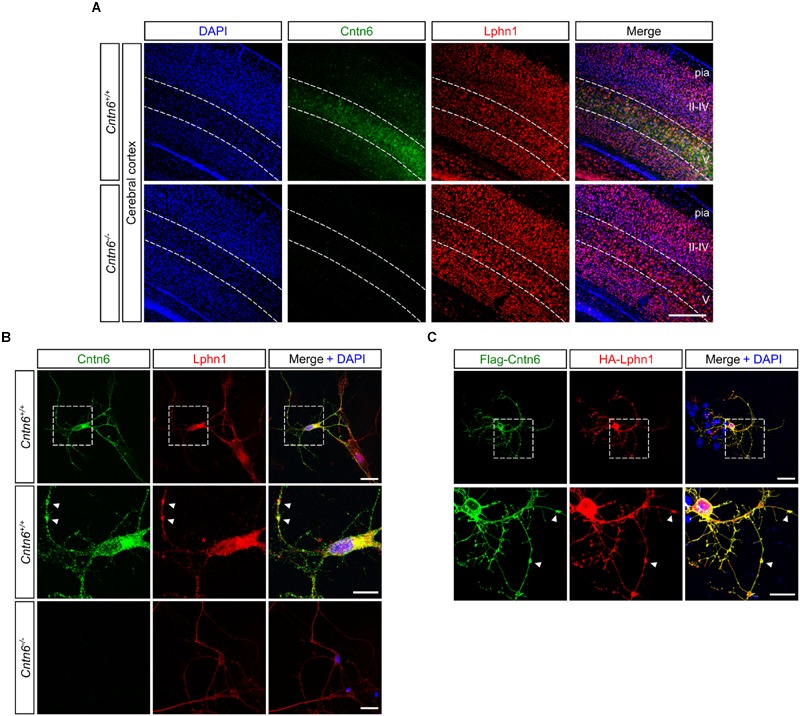
**Cntn6 and Lphn1 colocalize in the cortex. (A)** Immunostaining of Cntn6 (green) and Lphn1 (red) in wild-type and *Cntn6^-/-^* P14 animals demonstrating the coexpression of the proteins in layer V of the mouse cerebral cortex. DAPI staining is in blue. Scale bars represent 250 μm. (**B**, upper panel) Endogenous Cntn6 (green) and Lphn1 (red) colocalization is visible in wild-type primary cultures as opposed in *Cntn6^-/-^* primary cultures (**B**, bottom panel). DAPI staining is in blue. The scale bars indicate 20 μm. Middle panel show higher magnifications of boxed areas from the upper panel. The arrows indicate sites where the signals of Cntn6 and Lphn1 are clustered on neurites. The scale bar indicates 10 μm. **(C)** Cortical neurons were cotransfected with Flag-Cntn6 (green) and HA-Lphn1 (red) expression plasmids and immunostained after fixation. DAPI staining is in blue. Colocalization is visible throughout the neuron, The scale bar in the upper panel represents 20 μm. Lower panel shows higher magnifications of the boxed areas in the upper panel, with arrows indicating sites where the signals of Cntn6 and Lphn1 are clustered on neurites. The scale bar indicates 10 μm.

An endogenous Cntn6-Lphn1 heteromeric protein complex in the post-synapse can only be present in neurons that coexpress both proteins. We therefore determined and compared the expression patterns of *Cntn6* and *Lphn1*. First, the temporal expression of *Cntn6* was determined in the developing mouse brain by real time PCR. These experiments showed that *Cntn6* expression was highest at P7 (Supplementary Figure 2A), when *Lphn1* expression was also appreciable (Boucard et al., 2014). Comparison of *Cntn6* and *Lphn1* expression by *in situ* hybridization at P7 showed that *Cntn6* expression was more restricted as compared to *Lphn1* expression. Overlapping expression of both genes was particularly prominent in layer V of the cerebral cortex, the anterodorsal (AD) and anteroventral (AV) nuclei of the thalamus and the internal and external granular layers of the cerebellum (IGL and EGL respectively) (Supplementary Figure 2B). Additional overlapping expression was observed in layer V of the cerebral cortex, the subiculum and the CA1 region of the hippocampus. Immunostaining for Cntn6 and Lphn1 proteins confirmed the presence of both proteins in layer V of the cerebral cortex (**Figure 3A**) and in the AD and AV nuclei of the thalamus in P14 mice (Supplementary Figure 2C). No Cntn6 immunoreactivity was observed in *Cntn6^-/-^* mice, confirming the specificity of the immunohistochemical staining. Primary neuronal cultures immunostained for endogenous Cntn6 and Lphn1 proteins revealed colocalization at the cell surface and intracellular sites in neurons (**Figure 3B**). Staining along neurites was especially pronounced. Similar results were obtained by cotransfection with Flag-Cntn6 and HA-Lphn1 expression plasmids (**Figure 3C**).

Taken together, these data show that specific regions in the brain contain neurons that coexpress Cntn6 and Lphn1, suggesting that interaction of these proteins may serve biologically relevant functions. The coexpression in cortical neurons provided us with the opportunity to further examine these putative functions.

### Cntn6 Reverses Morphological Defects Induced by Lphn1

**FIGURE 4:**
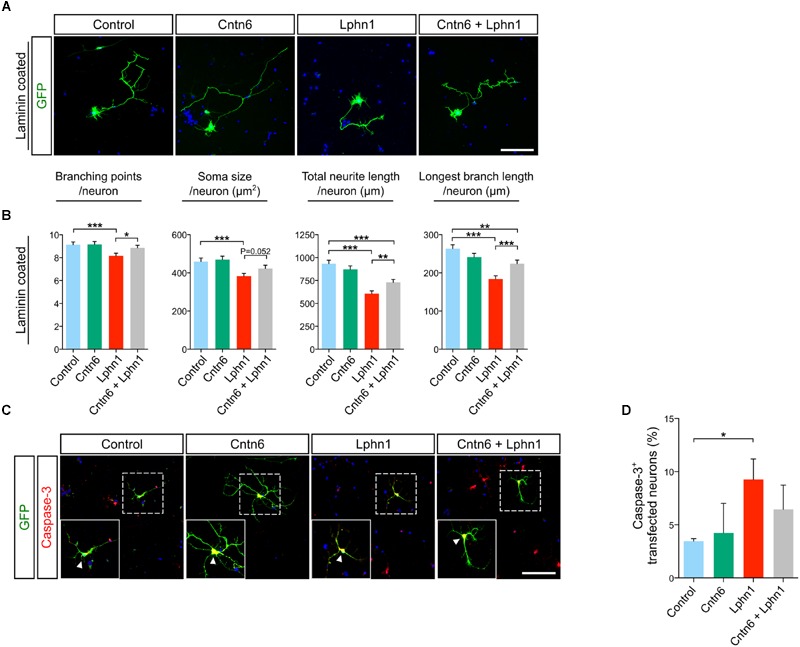
**Neuronal morphology of cortical neurons overexpressing Cntn6, Lphn1 or in combination. (A)** Mouse cortical cultures were transfected with Cntn6, Lphn1, Cntn6 + Lphn1 and control plasmids. Cultures were immunostained with an anti-GFP antibody (green). DAPI staining is in blue. **(B)** Morphological parameters of neurons cultured on Laminin were quantified and revealed a significant decrease on all parameters of the Lphn1 overexpressing neurons. **(C)** Representative photographs of caspase-3 (red) positive cells in neuronal cultures transfected with an EGFP expression vector (green) and vectors either for Cntn6, Lphn1, combination of both or an empty control plasmid. DAPI staining is in blue. Arrowheads indicate caspase-3 expression in the soma of neurons. **(D)** Apoptosis in neurons expressing Cntn6 and/or Lphn1 was quantified, showing an increase of apoptosis in Lphn1-expressing neurons. This was reduced by coexpression with Cntn6. About 60 transfected neurons were analyzed for caspase-3 immunoreactivity per condition of each independent experiment (*n* = 3). The scale bars indicate 100 μm. Statistical analyses were performed using unpaired Student’s *t*. The graph bars are presented as mean ± SEM. ^∗^*p* < 0.05, ^∗∗^*p* < 0.01, ^∗∗∗^*p* < 0.001.

To explore functions of the *cis*-interaction of Cntn6 and Lphn1, we investigated the effects of Cntn6 and Lphn1 expression alone and in combination, on the cellular morphology of cultured cortical neurons (**Figure 4A**). Cellular analysis showed that expression of Cntn6 did not affect neuronal morphology, including length and branching of neurites and soma size (**Figure 4B**). However, Lphn1-expressing neurons displayed a significant reduction in the number of branching points, soma size, total neurite length and longest branch length per neuron (**Figure 4B**). A progressive decrease of these parameters was observed with increasing plasmid concentrations (Supplementary Figure 3). Total neurite length and the longest branch length were most affected. Staining for the apoptosis marker caspase-3 showed that the level of apoptosis significantly increased upon expression of Lphn1 (**Figures 4C,D**). Markedly fewer transfected cells were observed in neuronal cultures transfected with Lphn1 overexpression plasmids (data not shown). This suggested that cultured neurons were severely compromised by Lphn1 expression and underwent apoptosis, pointing to a neurotoxic effect of Lphn1.

Notably, in these experiments coexpression of Cntn6 together with Lphn1 significantly reversed morphological parameters affected by Lphn1 alone (**Figure 4B**). When Cntn6 was coexpressed with Lphn1, morphological parameters reached or increased toward the levels in controls and neurons transfected with Cntn6 alone. Furthermore, the increased number of apoptotic neurons induced by Lphn1 expression was reduced by coexpression of Cntn6 (**Figures 4C,D**) Comparing Lphn1-expressing neurons cultured on Cntn6-coated substrate, providing Cntn6 in *trans*, or control BSA-coated substrate showed that Cntn6 in *trans* did not affect the neurotoxic effect of Lphn1 (Supplementary Figure 4). From these experiments we concluded that Lphn1 expression in cultured cortical neurons confers a neurotoxic activity resulting in altered overall neuronal morphology, reduced survival, which is rescued when Cntn6 interacts in *cis* with Lphn1.

**FIGURE 5:**
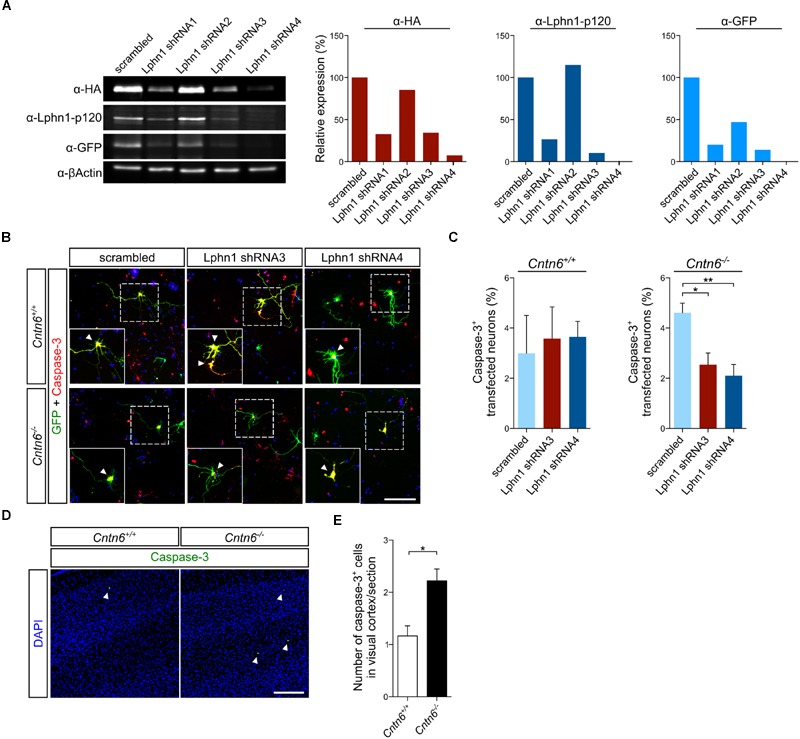
**Expression of Lphn1 in neurons results in increased apoptosis. (A)** Western blot of lysate from HEK293 cells that were contransfected with HA-Lphn1-GFP and one of four different Lphn1 shRNA plasmids or scrambled control plasmid (*n* = 1). The samples in these western blots that were immunoblotted with anti-HA, anti-Lphn1-p120, and anti-GFP antibodies were quantified and normalized against actin and the scrambled control plasmid, supporting strongest Lphn1 knockdown with the Lphn1 shRNA1, -3 and -4 plasmids. Molecular weights are as follows: HA-Lphn1-GFP = 125 kDa; βActin = 42kDa. **(B)** Representative photographs of caspase-3 (red) positive cells in wild-type and *Cntn6^-/-^* neuronal cultures transfected with an EGFP expression vector (green) and either a Lphn1 shRNA3 or -4 plasmid or a scrambled control plasmid. DAPI staining is in blue. Arrowheads indicate caspase-3 expression in the soma of neurons. The scale bar indicates 100 μm. **(C)** Cotransfection of Lphn1 shRNA3 and -4 plasmids with an EGFP expression vector in wild-type primary cultures did not result in apoptosis difference compared to the scrambled control plasmid. However, apoptosis in *Cntn6^-/-^* primary cultures was significantly reduced after treatment with Lphn1 shRNA3 and -4 compared to the scrambled control plasmid. About 60 transfected neurons were analyzed for caspase-3 immunoreactivity per condition of each independent experiment (*n* = 5 for *Cntn6^+/+^* cultures and *n* = 4 for *Cntn6^-/-^* cultures). **(D,E)** *In vivo* analysis of caspase-3 expression (green) in the visual cortex of P14 wild-type and *Cntn6^-/-^* animals revealed a significant increase of apoptosis in the absence of Cntn6. DAPI staining is in blue. Scale bar represents 150 μm. Analysis was performed on at least three sections per brain of wild-type and *Cntn6^-/-^* mice (*n* = 5 per genotype). Statistical analyses were performed using unpaired Student’s *t*. The graph bars are presented as mean ± SEM. ^∗^*p* < 0.05, ^∗∗^*p* < 0.01.

To further validate this interaction, Lphn1 knockdown was performed in primary cultures obtained from *Cntn6^-/-^* mice. Several short hairpin RNA (shRNA) expressing plasmids directed at different sequences of Lphn1 coding region were designed. Of these, shRNA3 and -4 were most efficient in reducing Lphn1 protein expression (**Figure 5A**). ShRNA3, -4, and the scrambled shRNA plasmids were separately transfected, together with EGFP, in primary cortical cultures derived from wild-type and *Cntn6^-/-^* mice. Immunostaining for EGFP and caspase-3 revealed that *Cntn6^-/-^* cortical cultures displayed an increase in apoptosis compared to wild-type cultures when transfected with the scrambled shRNA plasmid. When cortical cultures were transfected with the Lphn1 shRNA plasmids, apoptosis was significantly decreased in the *Cntn6^-/-^* cultures whereas this remained unchanged in the wild-type cultures (**Figures 5B,C**). These data indicated that endogenous Lphn1 was responsible for the increased level of apoptosis in *Cntn6^-/-^* neurons, and consequently that Cntn6 plays an endogenous role in inhibiting Lphn1-incuded apoptosis in these cultured neurons.

To critically test this indication *in vivo* apoptosis was assessed in the visual cortex of 14-day-old wild-type and *Cntn6^-/-^* mice. A significant increase of apoptosis was observed in the visual cortex of *Cntn6^-/-^* mice, compared to wild-type mice (**Figures 5D,E**). These results are in line with previous reports showing increased apoptosis in the internal granule cell layer of the cerebellum and in primary cultured cortical neurons in *Cntn6^-/-^* mice (Sakurai et al., 2009; Huang et al., 2011a). Taken together, the *in vitro* and *in vivo* data indicate that in the absence of Cntn6 Lphn1-induced apoptosis occurs. These data also indicate that the Cntn6-Lphn1 *cis*-complex is functional in controlling apoptosis.

## Discussion

Since *CNTN6* has been implicated in neurodevelopmental disorders we set out to examine pathways of action of this contactin member. Current insights in the mode of action of Cntn6 were too limited to fully explain its involvement in developmental mechanisms, despite the description of phenotypes in null mutant mice, such as biochemical interactors (Zuko et al., 2013). These phenotypes included a developmental delay of the corticospinal tract, a misorientation of apical dendrites in the cortex, altered numbers of subtype specific projection neurons and interneurons in the cortex, and an increase in neuronal cell death during cerebellar development (Ye et al., 2008; Sakurai et al., 2009; Pinto et al., 2010; Huang et al., 2011b; Zuko et al., 2016). Also, significant reduction in glutamatergic synapses was found in the hippocampus and in the cerebellum of *Cntn6* null-mutants (Sakurai et al., 2009, 2010). These data suggested that loss of *Cntn6* impairs development. Here we show that null-mutation of *Cntn6* increases apoptosis *in vitro* and *in vivo* and that this effect involves a pathway that is dependent on the presence of the adhesion GPCR Lphn1. Expression of Lphn1 in primary cultures resulted in smaller neurons, shorter neurites and increased apoptosis. These phenotypes were rescued by coexpression with Cntn6, indicating that Cntn6 inhibits these adverse effects of Lphn1. Moreover, Lphn1 knockdown in cultured neurons from *Cntn6^-/-^* mice reduced the amount of caspase-3-positive neurons, suggesting that Lphn1 indeed contributed to the increase of apoptosis in absence of Cntn6. Essentially this conclusion was supported by our *in vivo* findings of increased apoptosis in the visual cortex, a region coexpressing the *Cntn6* and *Lphn1* genes.

In line with this, it was previously reported that cortical cultures from *Cntn6^-/-^* animals displayed a significant decrease in cell survival (Huang et al., 2011a). Additionally, *Cntn6^-/-^* mice showed aggravated brain damage after trauma compared to wild-type mice, due to impaired neuronal survival and neurite growth (Huang et al., 2011a). Also the delay of corticospinal tract formation in *Cntn6^-/-^* mice might be attributed to the loss of a protective influence exerted by Cntn6 (Sakurai et al., 2009; Huang et al., 2012). These observations agree with a role of Cntn6 in preventing neurotoxicity.

The finding that Cntn6 reverses the effects of Lphn1 on neuronal morphological parameters and survival, only when coexpressed with Lphn1 and not when provided in *trans*, underscores the functional role for a Cntn6-Lphn1 heterodimeric *cis*-complex. This mechanism bears similarities to the concept of dependence receptors (Mehlen and Bredesen, 2004; Goldschneider and Mehlen, 2010; Mehlen and Tauszig-Delamasure, 2014). Cells expressing dependence receptors require the presence of a ligand to survive (Goldschneider and Mehlen, 2010; Mehlen and Tauszig-Delamasure, 2014). We show that Cntn6 is an endogenous ligand for Lphn1 and prevents this receptor to confer apoptosis when Cntn6 is present. As such Lphn1 may be considered a dependence receptor. Dependence receptors generally trigger two opposite signaling pathways depending on the occupation by their ligands. Classical signaling pathways are activated when bound to their ligands, supporting cell survival, migration and differentiation, and apoptotic signaling is conferred in unbound state (Goldschneider and Mehlen, 2010). As yet about 20 dependence receptors have been labeled as such, none of them belonging to the class of adhesion GPCRs (Mehlen and Tauszig-Delamasure, 2014). Adhesion GPCRs are a specific subfamily of receptors (Langenhan et al., 2013; Hamann et al., 2015; Krishnan and Schiöth, 2015) that display multiple signaling properties depending on structural conformation and state (Kenakin, 2011; Kishore et al., 2016). Indeed it has been reported that Lphn and Lat1, the *Caenorhabditis elegans* ortholog of mammalian Lphns, can activate Ca^2+^ and cAMP and bind to multiple G-proteins, suggesting that Lphns can activate multiple signal transduction cascades (Silva et al., 2011; Boucard et al., 2012; Müller et al., 2015).

The complex cell biology of adhesion GPCRs at the level of intracellular transport, proteolysis, reassociation, and dimerization and (self)activation (Langenhan et al., 2013; Hamann et al., 2015) leaves multiple possibilities for inhibition of Lphn1 by Cntn6 in *cis*. In our experiments Cntn6 appeared to be bound to full length Lphn1, since the tryptic peptides found in mass spectrometry were derived from N- as well as C-terminal parts of Lphn1 (Supplementary Figures 5A,B), and in our coIP experiments both Lphn1 N- and C-terminal domains were coprecipitated together with Cntn6 (**Figure 1**). Since we found that no difference in the relative quantities of the intact protein and ectodomain of Lphn1 was present in cells with or without coexpression of Cntn6 (Supplementary Figure 5C), it is suggested that the expression of Cntn6 does not affect the autoproteolysis of Lphn1, and that other mechanisms are involved which are subject of future experiments.

Since the apoptotic activity of Lphn1 is regulated in a *cis*-complex with Cntn6, coexpression of *Cntn6* and *Lphn1* is required for this mode of action. We found an increase of caspase-3-positive cells in the visual cortex of *Cntn6^-/-^* animals. The cortex indeed appeared one of the brain regions that contain neurons coexpressing *Cntn6* and *Lphn1.* Another region with marked co-expression of *Cntn6* and *Lphn1* mRNA was the cerebellum, in particular the IGL (Supplementary Figure 2B). A significant increase of cell death has also been reported previously in the IGL of the cerebellum of *Cntn6^-/-^* animals (Sakurai et al., 2009). However, *Lphn1* is more widely expressed in the brain than *Cntn6*. Therefore, we speculate that other ligands may regulate Lphn1 activity in brain regions where Cntn6 is absent. Such ligands potentially include Lasso/teneurin-2, FLRT3, and Nrxn1. These proteins all form high-affinity trans-synaptic ligand-receptor pairs with Lphn1 with signaling capabilities, shaping synapse structure and regulating synaptic development and function (Silva et al., 2011; Boucard et al., 2012; O’Sullivan et al., 2012). On the other hand, Cntn6 is known to complex with other membrane proteins as well, including Ptpra, Ptprg, PTPσ, Notch, and Chl1 (Cui et al., 2004; Hu et al., 2006; Ye et al., 2008; Bouyain and Watkins, 2010; Zuko et al., 2011). A case in which cell adhesion proteins form super-complexes with competing components has recently been made for Lphn3 association with Flrt and UncD members at the structural and functional level (Jackson et al., 2016). In the protein interaction repertoire of Lphn1 other ASD gene products are known to be present, in particular, Nrxn1 which is a major ASD gene interacting with Nlgn1 and LRRTMs (Ichtchenko et al., 1995; de Wit et al., 2009). Thus, Cntn6 may also link to the Nrxn1-Nlgn1 pathway of autism through interaction with Lphn1.

Present and previous data have indicated that loss of function of Cntn6 can result in increased apoptosis. Apoptosis plays a crucial role during development of organisms and organs, including the brain (Meier et al., 2000; Hipfner and Cohen, 2004; Mehlen and Bredesen, 2004). Apoptosis has also been implicated in neurodevelopmental disorders, particularly schizophrenia, in which cases with severe reduction of neuron numbers have been found (Margolis et al., 1994; Jarskog et al., 2005). It has been suggested that neurodevelopmental disorders with complex genetic etiology and large numbers of risk genes, including ASD, may include cases that share apoptosis as a pathogenic pathway (Wei et al., 2014). Neuropathology on brain tissues of autistic subjects has revealed an increase in apoptosis in several brain areas (Sheikh et al., 2010a,b). Furthermore, changes in apoptotic and anti-apoptotic proteins in post-mortem brain tissue have been found, and interpreted as derangements in regulation of apoptosis in autism (Fatemi and Halt, 2001; Araghi-Niknam and Fatemi, 2003; Mahfouz et al., 2015). This suggestion is further emphasized by several genetic animal models of ASD which display increased caspase-3 activity (Yochum et al., 2008; Sakurai et al., 2009; Olczak et al., 2010; El-Ansary et al., 2012). The *Cntn6*-deficient mouse shares this phenotype. Together, our data define Cntn6 as a ligand for Lphn1 modulating its apoptotic activity by direct binding thereby impinging on neurodevelopment.

## Author Contributions

AZ, AO-A, AA, AH, AP, YS, RP, and JB designed experiments; AZ, HP, and RvD performed experiments; AZ, RvD, and RT analyzed results; AZ, BvdZ, RP, and JB wrote the paper; all authors edited and approved the manuscript.

## Conflict of Interest Statement

The authors declare that the research was conducted in the absence of any commercial or financial relationships that could be construed as a potential conflict of interest.

## Footnotes

1 www.crapome.org
