# Erratum: Association of Cell Adhesion Molecules Contactin-6 and Latrophilin-1 Regulates Neuronal Apoptosis

**DOI:** 10.3389/fnmol.2017.00006

**Published:** 2017-01-19

**Authors:** Frontiers Production Office

**Affiliations:** Frontiers Production OfficeLausanne, Switzerland

**Keywords:** autism, ASD, neurodevelopmental disorders, cell adhesion molecules, neuronal outgrowth, Cntn6, Lphn1, apoptosis

Reason for Erratum:

In the final publication, the first names of the last two authors have been shortened to initials only (R. J. Pasterkamp and J. P. H. Burbach). The names should appear as follows: R. Jeroen Pasterkamp and J. Peter H. Burbach.

The publisher apologizes for the error. This error does not change the scientific conclusions of the article in any way and the original article has been updated.

