# Supplementary material for: Association of Cell Adhesion Molecules Contactin-6 and Latrophilin-1 Regulates Neuronal Apoptosis
Source: Front Mol Neurosci. 2016 Dec 15;9:143. doi: 10.3389/fnmol.2016.00143 (PMC5156884; doi:10.3389/fnmol.2016.00143)
Supplement: Supplementary file 1 [file Supplementary_Figures.docx]

**SUPPLEMENTARY FIGURE 1**

**
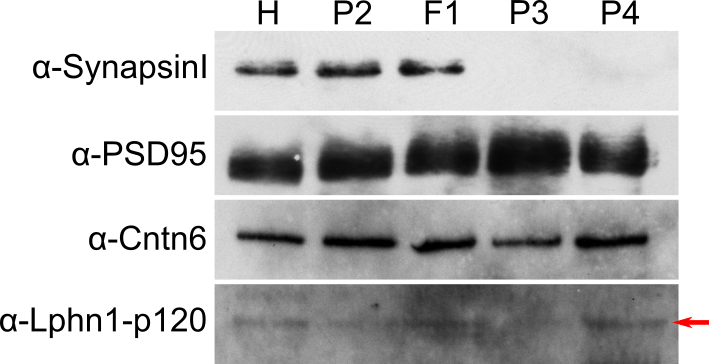
**

**SUPPLEMENTARY FIG. 1. SUBCELLULAR DISTRIBUTION OF CNTN6 AND LPHN1**. Subcellular fractionation revealed expression of Cntn6 and Lphn1 (arrow) together with markers of presynaptic fractions (SynapsinI) and postsynaptic fractions (PSD-95) in whole rat brain by means of Western blot. H = Homogenate; P2 = crude membrane fraction; F1 = synaptosomes; P3 = Triton-X100 insoluble postsynaptic fraction; P4 = postsynaptic density. Molecular weights are as follows: SynapsinI = 80kDa; PSD-95 = 100kDa; Cntn6 = 130kDa; Lphn1-p120 = 120kDa.

**SUPPLEMENTARY FIGURE 2**


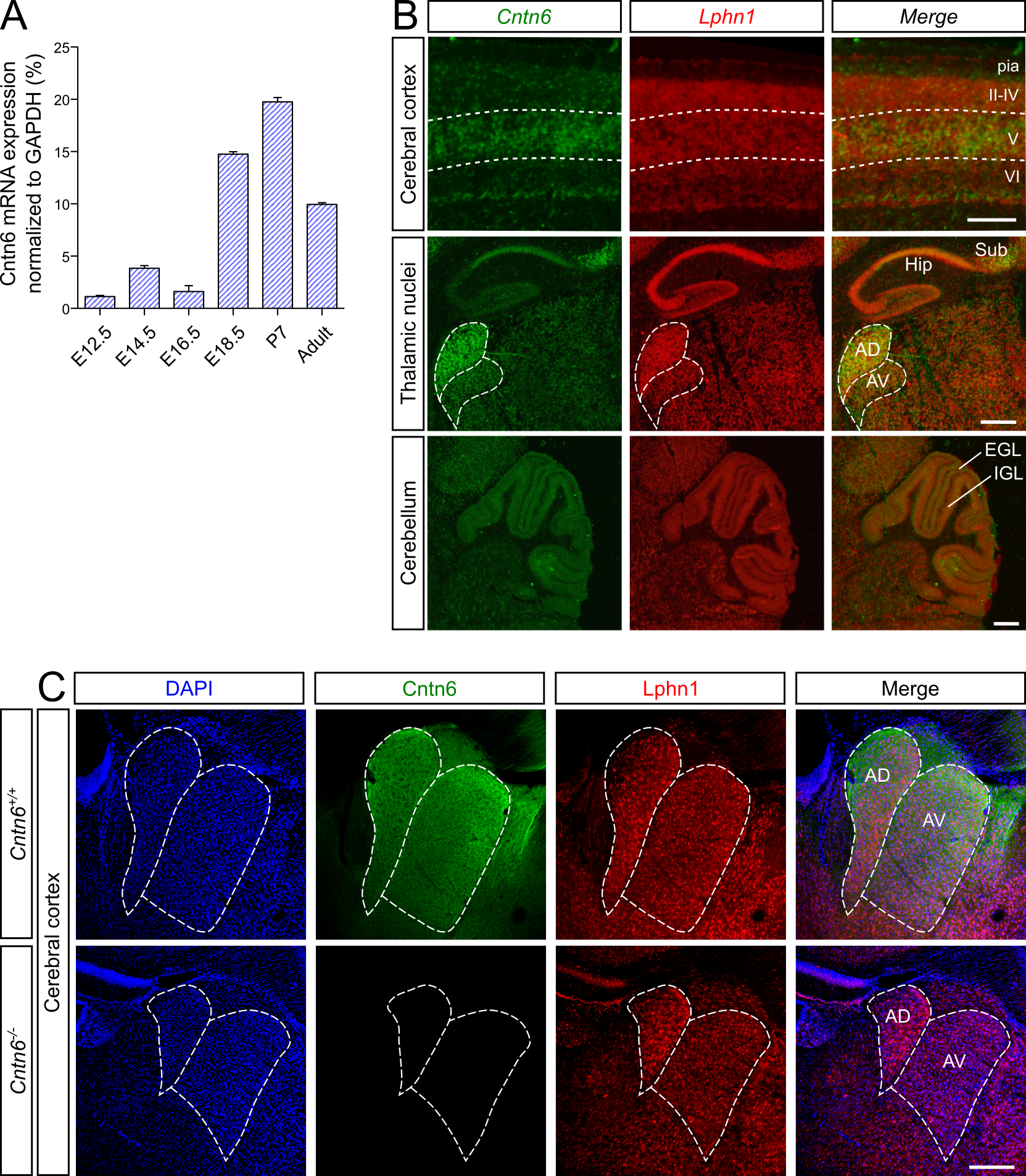


**SUPPLEMENTARY FIG. 2. CNTN6 AND LPHN1 COLOCALIZATION IN THE CORTEX.** (**A**) The expression of *Cntn6* reaches to peak levels at P7. Brain mRNA from different developmental changes was isolated, reverse-transcribed, subjected to qPCR to quantify *Cntn6* mRNA, which was normalized to *GAPDH* mRNA. (**B**) *In situ* hybridization showed coexpression of *Cntn6* (green) and *Lphn1* (red) in layer V mouse cerebral cortex, in the anterodorsal (AD) and anteroventral (AV) thalamic nuclei, hippocampus (Hip), subiculum (Sub), and in the internal and external granular layers of the cerebellum (EGL and IGL respectively) at P7. The scale bars represents 200μm. (**C**) Immunostaining of Cntn6 (green) and Lphn1 (red) in wild-type and *Cntn6^-/-^* P14 animals demonstrating the coexpression of the proteins in thalamic AD and AV nuclei. DAPI staining is in blue. Scale bars represent 250μm.

**SUPPLEMENTARY FIGURE 3**

**
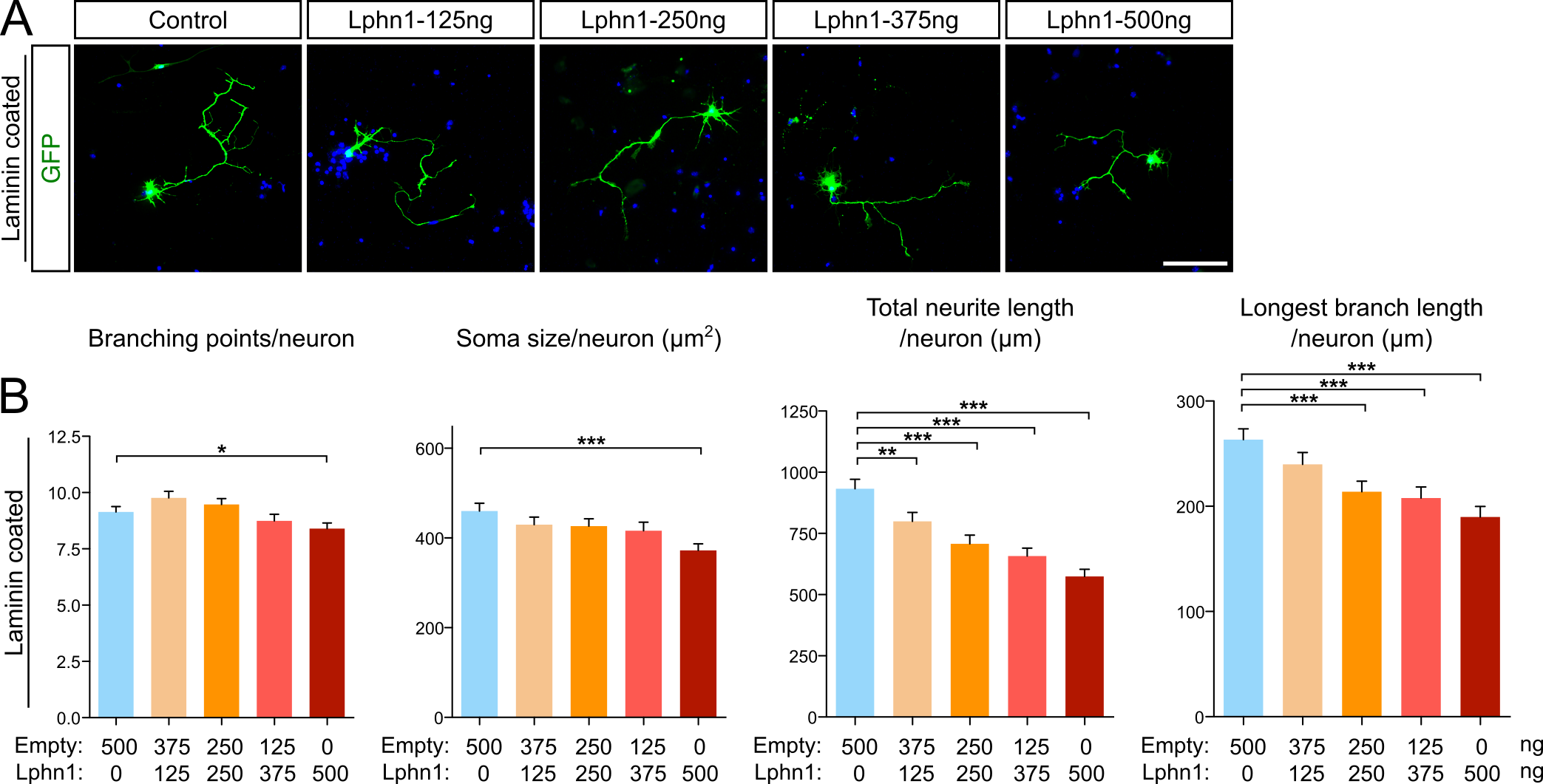
**

**SUPPLEMENTARY FIG. 3. NEURONAL MORPHOLOGY OF CORTICAL NEURONS OVEREXPRESSING AN INCREASING AMOUNT OF LPHN1.** (**A**) Representative images of neurons cotransfected with EGFP expression vector (green) and empty control plasmid or/and a Lphn1 plasmid concentration gradient varying from 120ng, 250ng, 375ng, and 500ng/well (of 12-wells plates). Cultures were fixed and immunostained with an anti-GFP antibody (green). DAPI staining is in blue. (**B**) Morphological parameters of neurons cotransfected with a Lphn1 plasmid concentration gradient revealed significant phenotypes corresponding with an increased Lphn1 plasmid concentration. Quantifications of morphological parameters were performed using Wis-Neuromath software. Parameters included the number of branching points, soma size, total neurite length and length of the longest neurite per neuron. Morphological analyses were performed of about 110 transfected neurons per condition of each independent experiment (n = 3).

**SUPPLEMENTARY FIGURE 4**


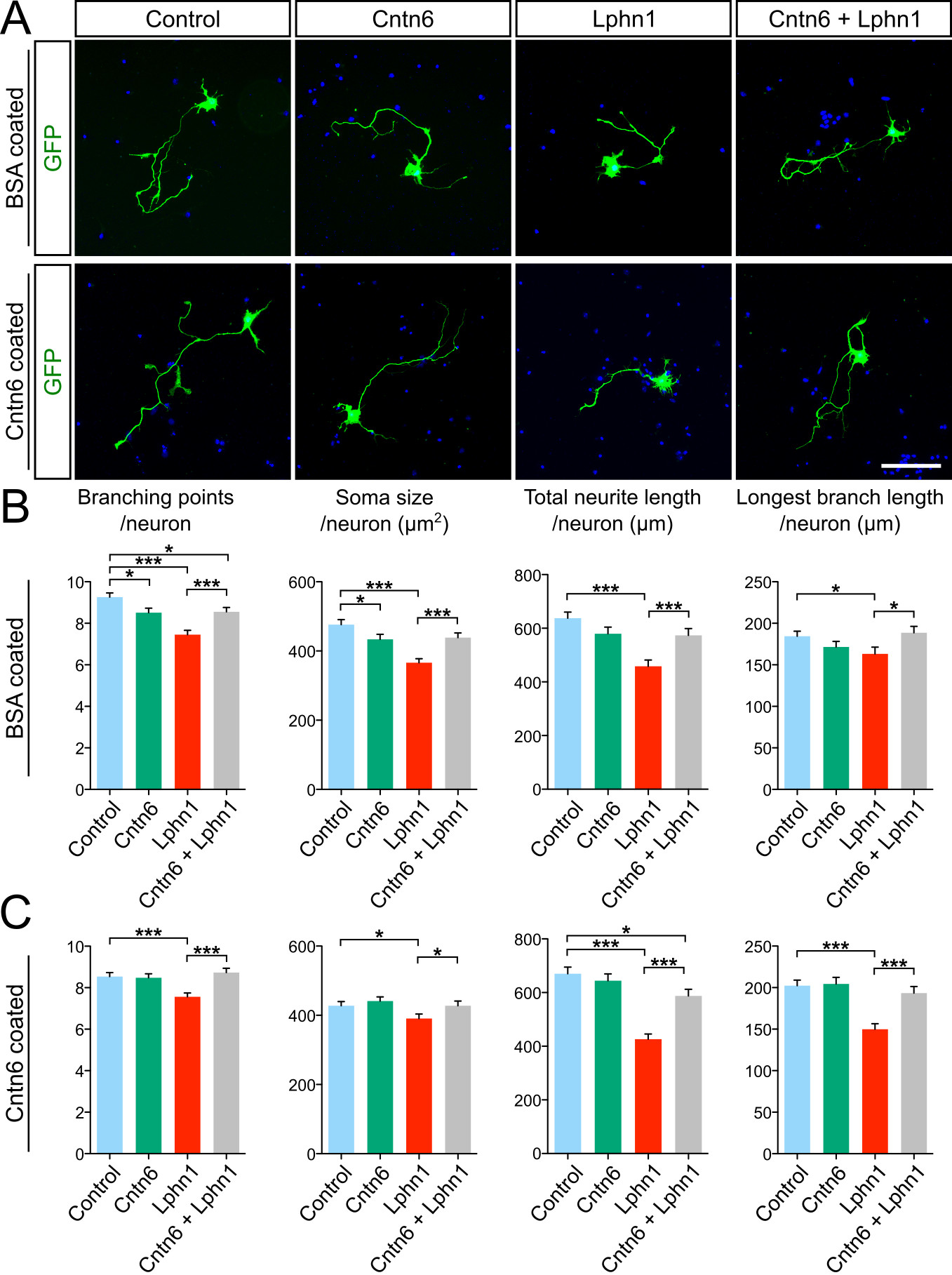


**SUPPLEMENTARY FIG.4. NEURONAL MORPHOLOGY OF CORTICAL NEURONS OVEREXPRESSING CNTN6, LPHN1 OR IN COMBINATION CULTURED ON SUBSTRATES.** (**A**) Mouse cortical cultures were transfected with Cntn6, Lphn1, Cntn6 + Lphn1 and control plasmids on Cntn6 and control BSA substrates. Cultures were immunostained with an anti-GFP antibody (green). DAPI staining is in blue. (**B-C**) Morphological parameters of neurons cultured on BSA or Cntn6 protein substrate were quantified and revealed no improvement on any parameters of the Lphn1 overexpressing neurons. Quantification of morphological parameters was performed using Wis-Neuromath software. Parameters included the number of branching points, soma size, total neurite length and length of the longest neurite per neuron. The scale bar indicate 100μm. Morphological analysis was performed of about 110 transfected neurons per condition of each independent experiment (n = 3). Statistical analysis was performed using unpaired Student’s *t* test and one-way ANOVA. The graph bars are presented as mean ±SEM. *, *p* < 0.05, **, *p* < 0.01, ***, *p* < 0.001.

**SUPPLEMENTARY FIGURE 5**


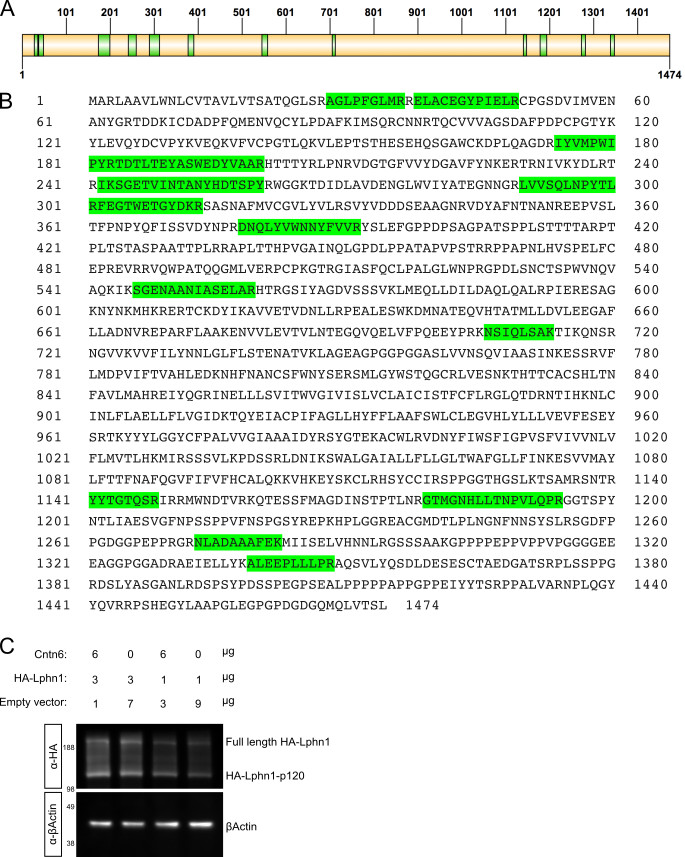


**SUPPLEMENTARY FIG. 5. CNTN6-LPHN1 INTERACTION DOES NOT PREVENT LPHN1 AUTOPROTEOLYSIS.** (**A-B**) Mass spectrometry of Cntn6-TMGFPBio IP eluates identified LPHN1 peptides across the entire LPHN1 protein (ACCESSION: NP_001008701). (**C**) HEK293 cells were cotransfected with Cntn6, HA-Lphn1 plasmids and an empty vector in different ratios that totaled 10μg. The lysates of these cells were analyzed by Western blot and immunoblotting with anti-HA antibodies revealed the intact form of HA-Lphn1 and the ectodomain of HA-Lphn1 (HA-Lphn1-p120). No difference was found in the amount of these domains in samples with and without Cntn6. Anti-βActin was used as an internal control. Molecular weights are as follows: Intact HA-Lphn1 = around 190 kDa; HA-Lphn1-p120 = 125kDa; βActin = 42kDa.
